# Supplementary material for: The Therapeutic Effect of Acanthopanax senticosus Components on Radiation-Induced Brain Injury Based on the Pharmacokinetics and Neurotransmitters
Source: Molecules. 2022 Feb 7;27(3):1106. doi: 10.3390/molecules27031106 (PMC8839712; doi:10.3390/molecules27031106)
Supplement: Supplementary file 1 [file molecules-27-01106-s001.zip › molecules-1575379-supplementary.pdf]

Table S1 The bioavailability of functional components in different tissues (%)

	**Polysaccharide (%)**	**Flavones (%)**	**Syringin (%)**	**EE (%)**
Liver	0.42 ± 0.02^{a}	0.15 ± 0.03^{b}	0.06 ± 0.02^{ab}	0.30 ± 0.03^{c}
Spleen	0.12 ± 0.01^{b}	0.05 ± 0.02^{b}	0.08 ± 0.04^{ab}	0.60 ± 0.04^{b}
Kidney	0.38 ± 0.02^{a}	0.01 ± 0.02^{d}	0.15 ± 0.05^{a}	1.01 ± 0.06^{a}
Testis	0.04 ± 0.01^{c}	0.03 ± 0.02^{b}	0.03 ± 0.01^{b}	0.06 ± 0.03^{d}
Heart	0.11 ± 0.00^{b}	0.02 ± 0.01^{c}	0.05 ± 0.02^{b}	0.52 ± 0.02^{b}
Brain	0.04 ± 0.01^{b}	0.10 ± 0.05^{a}	0.08 ± 0.03^{ab}	0.15 ± 0.02^{cd}

Vertical comparison of different letters in the upper right corner means significant difference ($P<0.05$, significant differences between the groups were tested by one-way analysis of variance)
